# A retrospective real-world study on semaglutide effectiveness in patients with obesity: differences between patients with and without food craving

**DOI:** 10.3389/fendo.2026.1927180

**Published:** 2026-10-12

**Authors:** Simona Bertoli, Federica Barbera, Chiara Cappellini, Alice Gotti, Alberto Battezzati

**Affiliations:** 1 IRCCS Istituto Auxologico Italiano, Obesity Unit and Laboratory of Nutrition and Obesity Research, Department of Endocrine and Metabolic Diseases, Milan, Italy; 2 International Center for the Assessment of Nutritional Status and the Development of Dietary Intervention Strategies (ICANS‐DIS), Department of Food, Environmental and Nutritional Sciences (DeFENS), University of Milan, Milan, Italy; 3 Postgraduate School of Clinical Nutrition, University of Milan, Milan, Italy; 4 IRCCS Istituto Auxologico Italiano, Clinical Nutrition Unit, Department of Endocrine and Metabolic Medicine, Milan, Italy

**Keywords:** body weight, food craving, obesity, real world evidence, semaglutide

## Abstract

**Background:**

Food craving (FC) is an established predictor of obesity. However, its role in weight management is insufficiently addressed in routine clinical practice.

**Methods:**

This retrospective study included adults with obesity, stratified by FC status, to compare the effectiveness of semaglutide plus hypocaloric diet (semaglutide group) versus hypocaloric diet alone (control group) in reducing body weight (BW). Assessments were conducted at baseline (T0), 3 months (T3), and 6 months (T6). The primary outcome was the between-group comparison of changes in BW from baseline at T3 and T6, also stratified by FC status. Secondary outcomes included changes in body mass index (BMI) and waist circumference (WC).

**Results:**

120 patients with obesity (mean age ± SD: 51.0 ± 15.9 years; 70% female) were included. Of these, 67 (56%) received semaglutide plus hypocaloric diet and 53 (44%) diet alone. FC was present in 53% of patients and was more frequent in women and younger individuals. BW, BMI, and WC significantly decreased in both semaglutide and control group over time (all p <0.0001). However, at 6 months, semaglutide group achieved greater reductions in BW (-3.3 kg; p=0.0004), BMI (-1.8 kg/m^2^; p=0.0015), and WC (-2.3 cm; p=0.03) compared with controls. Differences in BW reduction were maintained across patients with FC (-3.1 kg, p=0.03) and without FC (-3.4 kg; p=0.007).

**Conclusion:**

Semaglutide plus hypocaloric diet treatment is more effective than hypocaloric diet alone for weight reduction in real-world clinical practice, even in the presence of FC. Assessing FC may support more individualized obesity management strategies.

## Introduction

The term *craving* refers to an “urgent desire, longing, or yearning”, characterized by a compelling urge for a desired object that can reduce the ability to attend to stimuli unrelated to it (1). When this phenomenon pertains specifically to food, it is defined as *food craving* (FC), namely an intense desire to consume a particular food independent of physiological or metabolic need (2, 3).

In contrast to homeostatic eating, which is driven by energy requirements, FC is considered a component of hedonic eating associated with the pursuit of pleasure, and it has been identified as a significant predictor of both the development and maintenance of obesity (2–4). Despite this well-established association, FC is not routinely assessed or specifically targeted in clinical weight management, reflecting a potentially limited clinical interest in eating behaviour (3).

In our clinical practice, patients with obesity are managed through comprehensive clinical and behavioural characterization to optimize individualized and appropriate treatment approaches. In light of emerging evidence demonstrating that pharmacological therapies, such as semaglutide, reduce body weight (BW) and improve eating-related symptoms - including FC, preference for high-fat foods, and impaired eating control (5–8) - this therapeutic option is offered in combination with a tailored hypocaloric diet.

This preliminary study evaluated the effect of semaglutide combined with a personalized hypocaloric diet on BW in patients with obesity, stratified by the presence or absence of FC; moreover, it provided a real-world overview of the prevalence of FC among individuals attending our obesity treatment center. By presenting these preliminary observations, we hope to stimulate greater scientific interest in the assessment of eating behaviour and FC, and to encourage the scientific and clinical community to consider these factors more systematically when evaluating patients with obesity, ultimately promoting their integration into both obesity research and routine clinical practice.

## Methods

This was a real-world, single-center, controlled, retrospective study with the primary objective of evaluating the effect of semaglutide treatment combined with a personalized hypocaloric diet (semaglutide group) on BW in patients with obesity, with and without FC, over a six-month period. Outcomes were compared with those of patients following a hypocaloric diet alone (control group). Secondary objectives included within-group and between-group comparisons of body mass index (BMI) and waist circumference (WC).

The personalized hypocaloric diet was prescribed with an energy deficit of approximately 1000 kcal/day relative to individual energy requirements. Energy needs were estimated using the Mifflin–St Jeor equation (9) to calculate basal metabolic rate, multiplied by the individual physical activity level. Although most patients were eligible for semaglutide treatment, some declined pharmacological therapy for personal reasons and therefore followed a hypocaloric diet alone, constituting the control group. In the semaglutide group, treatment was initiated at a weekly dose of 0.25 mg and titrated every four weeks to reach the maintenance dose, in accordance with the Summary of Product Characteristics (SmPC) (10).

Data were extracted from medical records of consecutive patients at three predefined time points: baseline (T0), corresponding to treatment initiation (either semaglutide plus diet or diet alone), and two follow-up visits at approximately 3 months (T3; 1.5 ≤ T3 ≤ 4.5 months) and approximately 6 months (T6; 4.5 < T6 ≤ 7.5 months).

Patients were stratified according to the presence of FC at baseline. FC status was defined through structured interviews conducted during baseline visits by the same two trained physicians using a predefined script to ensure procedural consistency and minimize inter-observer variability. Participants were asked the following standardized questions: i) “*Do you experience recurrent and intense desires to eat specific foods, even in the absence of physical hunger?*”; ii) “*How frequently do these episodes occur?*” (<1 episode/week; ≥1 episode/week); iii) “*Are these episodes typically triggered by emotional states (e.g., stress, anxiety, sadness, or boredom)*?”. Definitions and classification criteria were established *a priori* to ensure reproducibility and transparency. Specifically, FC was defined as a recurrent, intense desire to consume specific foods, perceived as difficult to resist and not exclusively driven by physiological hunger. Clinically relevant FC was defined as episodes occurring at least once per week (≥1 episode/week) accompanied by perceived difficulty resisting the urge.

Quantitative variables were described using mean and standard deviation (SD), while categorical variables were described using absolute frequencies and percentages. Baseline difference between groups were assessed using Student’s t tests for continuous variables and Chi-squared tests for categorical variables. Linear mixed-effect models were used to evaluate changes in BW, BMI, and WC between baseline and follow-up (T3 and T6) and to assess the potential moderating effect of baseline FC (only for BW). The model included a random effect of subject to account for individual variability and was adjusted for sex and age at baseline. Models were fitted by restricted maximum likelihood using all available observations from participants with complete baseline covariate information, under the missing-at-random assumption; no multiple imputation was performed. To assess potential attrition bias at T6, baseline age, sex, FC, and the corresponding outcome were compared between completers and non-completers within each treatment group using Student’s t tests or Chi-squared tests, as appropriate. All p-values were adjusted for multiple comparisons using the Holm–Bonferroni method. Significance was set at p<0.05.

The study was conducted in compliance with the Declaration of Helsinki and ICH-Good Clinical Practice. Written informed consent was obtained from each participant (≥18 years) or, for minors, from their parents or legal representatives. Ethical approval was obtained from the assigned Ethical Committee (Approval no. 2022_11_22_04, date 22nd November 2022).

Given the retrospective observational design, no *a priori* sample size calculation was performed. The sample comprised all consecutive patients meeting the eligibility criteria during the predefined study period.

## Results

Data from 120 patients (mean age ± SD: 51.0 ± 15.9 years; 70% female) were included in the analysis. Of these, 67 patients (56%) were treated with semaglutide combined with a hypocaloric diet, while 53 (44%) followed a hypocaloric diet alone. Overall, 64 patients (53%) reported FC, including 42 in the semaglutide group and 22 in the control group.

Additional demographic and clinical baseline characteristics are reported in **Table 1**.

**Table 1:** Baseline characteristics of the study population overall and stratified by treatment group (Semaglutide vs Control) and between-group differences in BW mean change from baseline, stratified by absence or presence of FC.

Variable	Value	Overall (N = 120)	Semaglutide group (N = 67)	Control group (N = 53)	P value semaglutide vs control
Age, years	*Mean (SD)*	51.0 (15.9)	50.8 (15.7)	51.4 (16.3)	0.86
Sex, n (%)	*F*	84 (70%)	52 (78%)	32 (60%)	0.07
	*M*	36 (30%)	15 (22%)	21 (40%)	
BW (kg)	*Mean (SD)*	90.0 (15.1)	90.7 (14.3)	89.1 (16.0)	0.56
BMI (kg/m^2^)	*Mean (SD)*	33.0 (5.0)	33.2 (4.5)	32.8 (5.6)	0.70
WC (cm)	*Mean (SD)*	105.4 (12.4)	104.9 (11.7)	106.3 (13.5)	0.53
FC, n (%)	*Yes*	64 (53%)	42 (63%)	22 (42%)	**0.03**
	*No*	56 (47%)	25 (37%)	31 (58%)	
Between-group differences (Semaglutide vs Control) in BW mean change from baseline according to FC					
FC	Time-point	Between-group difference in BW mean change from baseline (kg) *^a^*	95% CI (lower, upper)	p value	adjusted p value
No	T3	-1.3224	-3.2343, 0.5896	0.17	0.17
	T6	-3.4000	-5.6466, -1.1534	**0.003**	**0.007**
Yes	T3	-0.5457	-2.4274, 1.3360	0.57	0.57
	T6	-3.1092	-5.6283, -0.5902	**0.016**	**0.03**

Data are presented as mean (SD or 95%CI) or n (%). Percentages are calculated on the total N of each column. ^a^Values represent the difference in mean BW change from baseline between the semaglutide and control groups. Negative values indicate greater BW loss in the semaglutide group. Statistically significant p values (p<0.05) are indicated in bold. SD, standard deviation; BW, body weight; BMI, body mass index; WC, waist circumference; FC, food craving; CI, confidence interval.

Patients with FC were significantly younger compared with those without FC (mean age ± SD: 47.9 ± 14.7 vs 54.7 ± 16.6 years, respectively; p=0.018) and FC was significantly more frequent among female patients (patients with FC: 80% female vs 20% male; patients without FC: 59% female vs 41% male; p=0.02).

BW significantly decreased from baseline to T3 and T6 in both the semaglutide and control groups (all p<0.0001). At T6, patients treated with semaglutide experienced significantly greater weight loss compared with those in the control group (mean difference: −3.3 kg; p=0.0004) (**Figure 1A**). This between-group difference was maintained regardless of the presence of FC: at T6, semaglutide-treated patients lost significantly more BW than controls both in patients with FC and in those without FC. Although slightly less statistically significant in individuals with FC, the between-group BW loss exceeded 3 kg in both patients with and without FC (**Table 1**).

**Figure 1:**
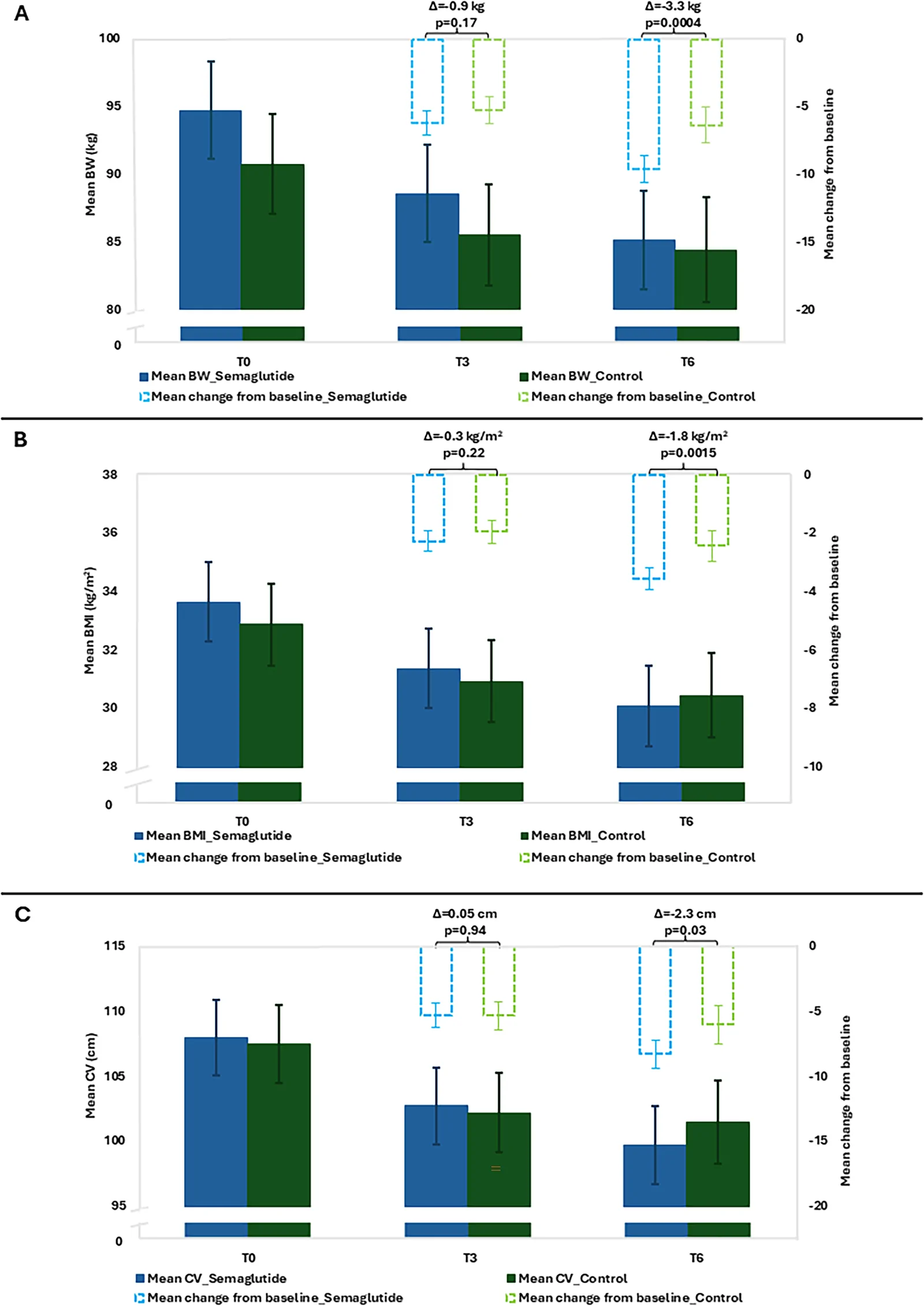
BW **(A)**, BMI **(B)**, and WC **(C)** changes over time in the semaglutide and control groups. Solid bars represent mean BW (left y-axis) at baseline (T0), 3 months (T3), and 6 months (T6) in the semaglutide and control groups, with error bars indicating the 95% CI. Significant within-group reductions in BW, BMI, and WC were observed from baseline to T3 and T6 in both groups (all p<0.0001). Dashed bars represent the mean change (right y-axis) from baseline to T3 and T6, with error bars indicating 95% CI. Between-group differences (Δ; Semaglutide vs Control) in mean BW change from baseline are shown with corresponding p values. BW observations: semaglutide group - T0 n=65, T3 n=59, T6 n=47; control group - T0 n=53, T3 n=45, T6 n=24. BMI observations: semaglutide group - T0 n=64, T3 n=59, T6 n=47; control group - T0 n=53, T3 n=45, T6 n=24. WC observations: semaglutide group - T0 n=66, T3 n=58, T6 n=45; control group - T0 n=53, T3 n=42, T6 n=24. Abbreviations: BW, body weight; BMI, body mass index; WC, waist circumference; CI, confidence interval.

BMI and WC also significantly decreased at T3 and T6 compared with baseline in both the semaglutide and control groups (all p<0.0001). However, at 6 months, patients treated with semaglutide showed significantly greater reductions in BMI (p=0.0015) and WC (p=0.03) compared with controls (**Figures 1B, C**).

Attrition analyses comparing participants with and without a 6-month assessment showed some descriptive differences in baseline characteristics; however, none reached statistical significance within either the treated or control group for body weight, BMI, or waist circumference (all p>0.05). Thus, no statistical evidence of an association between loss to follow-up and the measured baseline characteristics was observed, although the relatively small sample sizes limit the power of these comparisons.

## Discussion

This study demonstrated the greater effectiveness of semaglutide combined with a hypocaloric diet compared with a hypocaloric diet alone in reducing BW, BMI, and WC over a 6-month period in patients with obesity in a real-world setting, confirming previous evidence from clinical practice (11, 12). The novel aspect of this study is the stratification of patients according to FC status at the baseline. Notably, patients treated with semaglutide showed greater reductions in BW compared with controls, independently of FC status. The between-group difference in BW change indicated a clinically meaningful benefit of semaglutide regardless of craving status: indeed, six months after treatment initiation, patients receiving semaglutide associated with hypocaloric diet showed a greater mean weight loss than controls, with differences of 3.1 kg in patients with FC and 3.4 kg in those without FC (both p<0.05).

An additional noteworthy aspect is that the semaglutide group included a higher proportion of patients with FC compared with the control group. This may reflect a greater propensity among patients with a long-standing history of difficulty losing weight to accept pharmacological treatment, whereas those in the control group - although eligible for semaglutide - may have experienced fewer weight management difficulties and therefore been less willing to incur the financial cost of pharmacological treatment. Importantly, when considering the unstratified comparison between semaglutide group and controls (**Figure 1A**), the semaglutide group - despite this higher proportion of patients with FC and thus a potentially more challenging clinical profile - still achieved a significantly greater reduction in BW at 6 months compared with controls (-3.3 kg; p=0.0004). Notably, these patients did not receive any structured psychological support, suggesting that pharmacological intervention may also contribute to the behavioural aspects of eating regulation. These effects have previously been demonstrated in controlled clinical settings, including the 104-week STEP 5 and 72-week STEP UP trials (7, 8), in which semaglutide, compared with placebo, significantly improved control over FC, reduced savoury and sweet cravings, improved mood, and ameliorated emotional and uncontrolled eating behaviours.

Interestingly, FC was more prevalent among younger individuals and women, suggesting a potential role of emotional and behavioural factors, particularly in these subgroups. These findings may have practical implications, as consideration of emotional factors, age, sex, and prior weight management history could help clinicians tailor individualized treatment strategies.

Despite providing novel insights, this study has several limitations. FC was assessed using a structured clinician-administered interview rather than a validated psychometric questionnaire, which limits comparability with studies using standardized instruments and may have introduced reporting bias. Treatment self-selection at baseline and unmeasured factors (i.e. socioeconomic status, individual treatment preferences, and motivation) may have resulted in residual confounding. FC status was not assessed at the follow-up. However, this remains an important domain for future prospective research, helping to clarify whether changes in FC contribute to weight-loss response with semaglutide.

The observational, retrospective, and non-randomized nature of the study may limit causal interpretation in between-group comparisons; therefore, these findings should be interpreted in the context of a real-world setting.

However, this report provides real word evidence and reflects the authors’ routine clinical practice where the use of validated instruments is often constrained by time and logistical limitations while structured interviews are commonly used to assess eating behaviours.

In conclusion, semaglutide treatment combined with a hypocaloric diet was more effective in reducing BW than a hypocaloric diet alone, including in patients with FC. Through this study, we sought to provide a preliminary real-world overview of the prevalence of FC and its distribution according to patient characteristics such as age and sex, factors that may deserve greater consideration during the clinical assessment of individuals with obesity. Although preliminary, these findings highlight the potential clinical relevance of assessing FC in routine practice, as it may inform therapeutic decision-making and help tailor treatment strategies according to individual behavioural and clinical phenotypes. By incorporating these behavioural dimensions into clinical evaluation, clinicians may be better equipped to address behavioural drivers of eating and, consequently, support patients in achieving more effective weight-loss outcomes. Ultimately, we hope this work will encourage greater attention to the behavioural aspects of obesity and promote the systematic assessment of FC in both research and clinical practice.

## Data Availability

The original contributions presented in the study are included in the article/supplementary material, further inquiries can be directed to the corresponding author/s.
